# Effect of 3D superimposition software on intraoral scanner accuracy and monitoring outcome

**DOI:** 10.1007/s00784-026-07136-8

**Published:** 2026-09-18

**Authors:** Albert Mehl, Jenny Buhl, Andreas Ender

**Affiliations:** Division of Computerized Restorative Dentistry, Center for Dental Medicine, University of Zurich, Plattenstrasse 11, Zurich, 8032 Switzerland

**Keywords:** Intraoral scanner, 3D alignment, ICP registration, Quantile‑based metrics, Accuracy

## Abstract

**Objectives:**

To determine whether 3D superimposition software (“Apps”) systematically affects 3D deviation outcomes derived from intraoral scanner (IOS) data, using two quantile‑range accuracy metrics (Q90_Q10_half and Q95_Q05_half).

**Materials and methods:**

Two jaw‑sized reference models were digitized with a high‑resolution optical scanner (ATOS III) to generate reference STL meshes (REF_STL). Five IOS devices acquired ten scans per device and per jaw model, exported as STL meshes (IMP_STL): Emerald Green, iTero 5D, Medit i900, Primescan, and Trios 5. Each IMP_STL was trimmed (1 mm above gingival line) and superimposed to REF_STL in four Apps: commercial industrial software: Geomagic Control X and GOM Inspect, and open dentistry-focused software Medit Design (Compare) and Dentexion; resulting in total to *N* = 400 superimpositions. For each superimposition, Q90_Q10_half and Q95_Q05_half values were computed from point-wise signed surface distances. A linear mixed‑effects model tested factor App as fixed effect with random intercepts for factors Device and Jaw, followed by Bonferroni‑adjusted pairwise comparisons (α = 0.05).

**Results:**

App significantly influenced Q90_Q10_half (*p* < 0.01) and Q95_Q05_half (*p* < 0.001). Control X yielded higher adjusted means than Dentexion and Compare for Q90_Q10_half (both *p* < 0.01), while Dentexion, GOM, and Compare did not differ. For Q95_Q05_half, Control X exceeded all other Apps (all *p* < 0.01). Notably, IOS rankings were stable across Apps, indicating that software altered magnitude but not relative ordering.

**Conclusions:**

Evaluation software is a significant source of variability in 3D deviation metrics; Control X produced consistently higher (more conservative) quantile‑range values, particularly for Q95_Q05_half.

**Clinical relevance:**

Inter‑study comparisons in digital accuracy research should report the evaluation software and settings. Dentistry‑focused Apps (e.g., with automated tooth segmentation) may reduce operator‑dependent effects and facilitate clinically applicable monitoring.

## Introduction

Intraoral scanners (IOSs) are integral to digital dentistry, enabling chairside acquisition of three-dimensional (3D) impressions for diagnostic, restorative, orthodontic, and implant workflows [1]. Their accuracy, defined by trueness and precision [2], is generally clinically acceptable, although deviations depend on scanner- and operator-related factors, such as scanning technology, strategy, operator experience, ambient lighting, and edentulous span, and also increase with scan length and anatomical complexity [3–10]. Research has mainly focused on these scanner- and operator-related factors.

Evaluation of IOS accuracy may also depend on the analysis software used for comparing 3D surface data. Typically, STL or PLY files from IOSs are superimposed onto a reference dataset using best-fit alignment algorithms (often ICP), followed by quantitative deviation analysis [9, 10]. Commonly reported metrics include root mean square (RMS) error, absolute mean distances, and quantiles. Among these, quantile-based measures have proven reliable for describing mesh coincidence after best-fit alignment, because they capture distributional behavior without being dominated by outliers [2, 11]. For alignment and deviation analysis, various software solutions are employed, notably commercial packages such as GOM Inspect and Geomagic Control X, as well as open-source tools like CloudCompare [12–15]. Recently, dentistry-focused platforms such as Medit Design (Compare) and OraCheck have emerged, offering improved user-friendliness compared to industrial-oriented software. While Compare supports export of statistical data for research purposes, OraCheck lacks functionality for exporting aligned meshes and associated statistics, limiting its suitability for mesh-to-mesh comparison studies [12, 16].

Evidence suggests that the choice of evaluation software represents a substantial and often underappreciated source of variability in IOS accuracy research. Son et al. demonstrated that identical datasets produced significantly different RMS values depending on the analysis software, particularly in half-arch and localized regions, whereas differences were less pronounced in complete-arch evaluations [13]. Complementary analyses indicate that algorithmic differences - especially in alignment strategies, surface sampling density, region handling, and outlier rejection - can influence deviation values to an extent comparable to differences between scanner systems [15]. To address some of these issues, Dentexion - a newer cloud-based solution - implements automated alignment and tooth-level segmentation tailored to intraoral scans. This enables consistent tooth- or region-based evaluations (e.g., per tooth or surface subset), aligned with recent advances in automated segmentation [17, 18]. 

Despite the awareness of these specific evaluation software factors, systematic comparisons under identical full-arch conditions remain limited, especially regarding histogram-based quantile metrics. This is clinically relevant, because full-arch assessments, commonly used to benchmark scanner performance, are susceptible to software-dependent alignment behavior [3, 12, 15]. Furthermore, post-superimposition metrics are increasingly used for monitoring and diagnostics and may also depend on the underlying software-dependent calculation methods. As software packages may apply different algorithmic strategies, the comparisons in this study should, in a first step, reflect software-specific differences rather than providing a fully standardized IOS accuracy measurement protocol.

Therefore, the aim of this study was to compare four evaluation software programs - GOM Inspect, Geomagic Control X, Medit Design (Compare), and Dentexion - with respect to their influence on quantile-based metrics used for defining the full-arch scan accuracy, while accounting for measurement variability across 5 IOS devices and two jaw models. The hypothesis was that, after adjusting for variability due to device and jaw, no differences exist in the quantile metrics produced by the four evaluation Apps.

## Materials and methods

### Study design and data acquisition

This in vitro study was conducted at the Center for Dental Medicine, University of Zurich, Switzerland. Two custom-made complete-arch models of a typical lower and upper jaw were utilized as reference models (group Jaw) [11]. The exact model fabrication and visualization have already been described in more detail in a former study [19].

First, the reference models were scanned using a high-resolution reference scanner (ATOS III, Triple Scan MV60; GOM). This approach has been shown to provide trueness of 3 μm and precision of 2 μm for jaw-sized scans [20]. The generated reference files were exported as a standard tessellation language (STL) file (REF_STL).

Digital scans of the reference models were subsequently obtained using 5 different IOSs (group Device): Emerald Green (EME; PLANMECA; Helsinki; Finland), iTero 5D (ITE; Align Technologies; San Jose; CA, USA), Medit i900 (MED; Medit; Seoul; South Korea), Primescan (PRI; Dentsply Sirona; Bensheim; Germany), and Trios 5 (TRI; 3Shape; Copenhagen; Denmark). All scans were obtained by two examiners, each with > 2 years’ experience. All strategies, including calibration and scanning, were in accordance with the manufacturer’s recommendations and described in Buhl et al. [11]. A total of 10 scans for each device were performed per jaw model (upper and lower jaw model). The jaw models were scanned without any coating. All scans were exported to STL files (IMP_STL) for further processing (*N* = 100 IOS scan data).

### Data processing and statistical evaluation

**Fig. 1:**
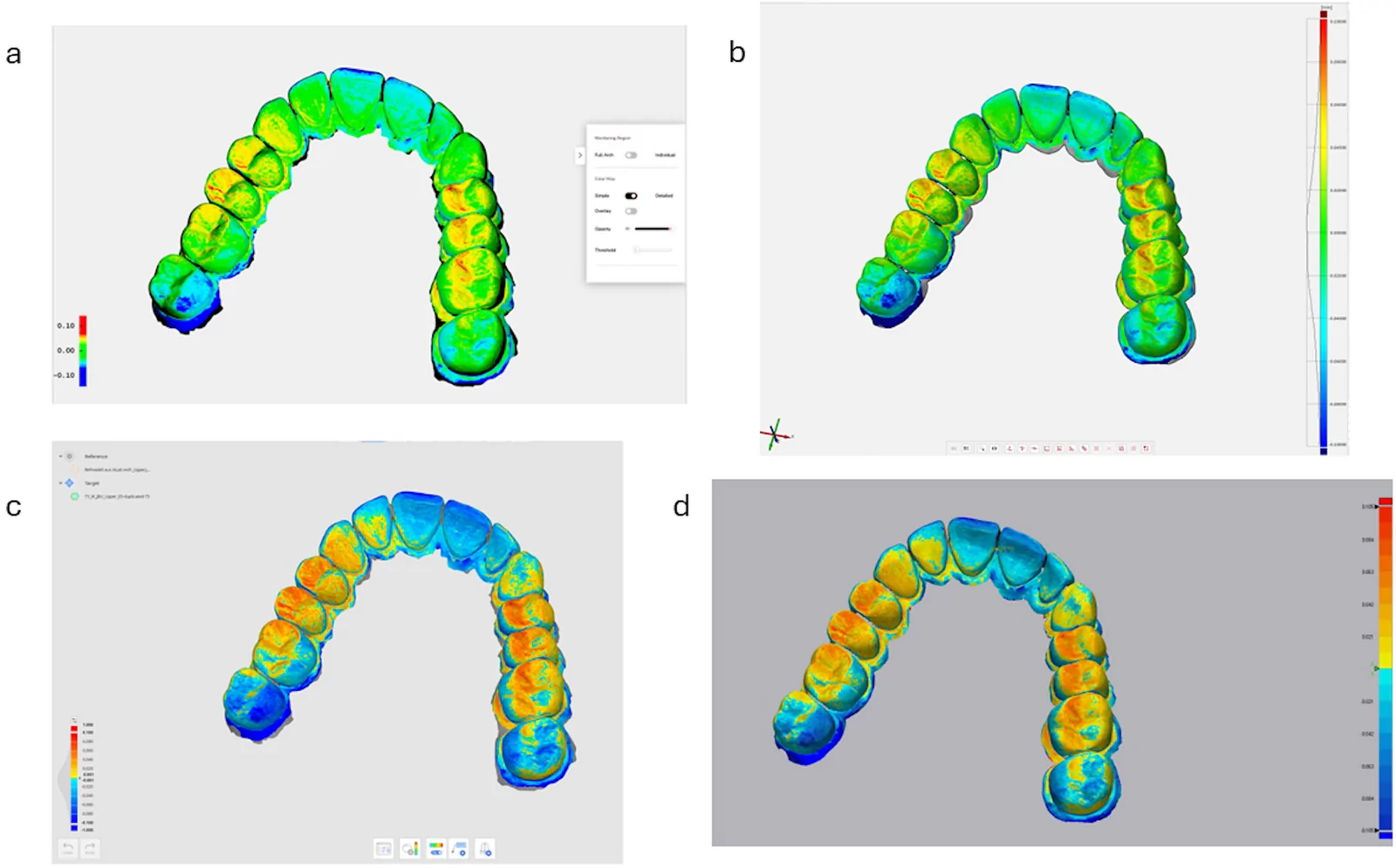
Example of the superimposition of the same IOS scan (IMP_STL) onto the reference data (REF_STL) using four different software evaluation programs (Apps): (**a**) Dentexion, (**b**) GOM Inspect, (**c**) Compare, and (**d**) Geomagic Control X. The signed‑distance pattern is nearly identical across all Apps; however, the color‑coded differences vary slightly due to App‑specific color scales and minor deviations in calculated distance values. For all Apps, the signed‑distance display range was limited to − 0.1 mm to + 0.1 mm

Each IMP_STL from the test groups was superimposed to the respective REF_STL set using 4 different programs (group App): Geomagic Control X ([ControlX]; Professional EDU, 2024.2.0, 3D Systems, Rock Hill, USA), Dentexion (Open Prototype Version, Dentexion, Zug, Switzerland), GOM Inspect 2018 ([GOM]; Carl Zeiss GOM Metrology, Braunschweig), Medit Design ([Compare], v. 2.1.4.97, Medit; Seoul; South Korea). Before alignment, all scan datasets were trimmed to a distance of 1 mm from the gingival margin to achieve a surface area comparable to that of the reference dataset (REF_STL) (Fig. 1). Furthermore, the same reference dataset was used as the baseline/reference (normal) mesh for all superimpositions in all 4 Apps. Together, these standardizations ensured that the superimposition was performed on essentially identical regions across all scan datasets. Additionally, it should be noted that no downsampling, mesh decimation, resampling, or any other data preprocessing was performed before importing the scans into the respective applications. Consequently, exactly the same scan datasets served as input for all four software packages, ensuring that any observed differences originated from the software-specific processing workflows rather than from differences in the input data.

After best-fit alignment in the respective Apps, signed 3D-distances were calculated for each surface point of the scan data mesh (IMP_STL) to the surface of the reference data mesh (REF_STL) (Fig. 1). This evaluation feature, i.e. signed point-to-triangular-surface distance calculation, was set in all 4 Apps. For each superimposition, the distance values were then exported into a readable text file (CSV) in case of GOM and ControlX, and from those distances, both the value (90% quantile – 10% quantile)/2 and the value (95% quantile – 5% quantile)/2 (i.e. Q90_Q10_half and Q95_Q05_half) were calculated as the respective accuracy metrics using SPSS software (version 30.0.0.0; IBM; Armonk, NY, USA). For the Apps Dentexion and Medit Compare, the quantile metrics were taken directly from the result reports of the respective Apps.

The process of data superimposition and evaluation for each App was in detail:

ControlX: import REF_STL and IMP_STL into the software. Define REF_STL as “Reference Data”. Perform “Initial Alignment” to superimpose the data sets. Perform a “Best Fit Alignment” to improve superimposition. Select “3D Compare” and select “Shortest” in “Calculation Options”-> “Export 3D Compare” results as CSV File.

Dentexion: Upload REF_STL and IMP_STL, add new media with ADD NEW, select Monitoring and choose REF_STL as Baseline and IMP_STL as Follow up, leave advanced default settings unchanged (especially keep the setting that all segmented teeth will be used for alignment), open the “View” for the superimposition result, and go to the “report” sheet. The data was then transferred via copy and paste to an excel sheet.

GOM: import REF_STL and IMP_STL, define REF_STL as “Nominal Element”, under menu item “Alignment” choose “Prealignment with Additional best-fit” and select the respective IMP_STL. After Alignment, go to “Compare” and “On mesh”, and then export the distance data into a CSV file.

Compare: import REF_STL and IMP_STL. Select “Alignment Mode”. Define REF_STL as reference data set. Define one IMP_STL as target set. Superimpose data with “Automatic Alignment”. Select “Deviation Display Mode”. Select measures in “Deviation Settings”. The measures are displayed at the lower left side of the program window. These calculated measures are exported as screenshot and manually transferred to an excel sheet.

Finally, for each scanning device and each evaluation program *n* = 20 accuracy metrics (2 jaws x 10 scans) were available for further statistical analysis (in total: *n* = 400 for both Q90_Q10_half and Q95_Q05_half metrics). No a priori sample size calculation was performed because no comparable published data were available that would have allowed a reliable estimation of the expected App-related effect size for the investigated quantile metrics. However, a post hoc power analysis was conducted based on the observed effects of the primary outcome measures to assess whether the achieved sample size provided sufficient statistical power.

All statistical analyses were performed using SPSS software (version 30.0.0.0; IBM; Armonk, NY, USA). The primary outcome variables were Q90_Q10_half and Q95_Q05_half, obtained from 10 repeated measurements for each combination of Device, Jaw, and App. Because measurements were nested within scanning devices and jaws, and because the study aimed to isolate the systematic effect of the evaluation application independently of these sources of variability, a linear mixed‑effects model (LME) was used.

In this model, App (ControlX, Dentexion, GOM, Compare) was specified as a fixed effect, representing the factor of interest. Device and Jaw were modeled as random intercepts to account for clustering and heterogeneity introduced by differences between scanning systems and anatomical regions. Model parameters were estimated using restricted maximum likelihood (REML), and denominator degrees of freedom for fixed effects were computed using the Satterthwaite approximation. The primary hypothesis - that the adjusted mean of Q90_Q10_half and Q95_Q05_half values do not differ among Apps - was tested using the Type III F‑test for the fixed effect of App. Statistical significance was defined as *p* = 0.05 (two‑sided). Variance components and intraclass correlation coefficients (ICCs) for Device and Jaw were extracted from the fitted model to quantify the relative contribution of these factors to total measurement variance. All assumptions for mixed‑effects modeling (linearity, normally distributed residuals, homoscedasticity across fitted values) were verified through visual inspection of diagnostic plots. Upon a statistically significant global App effect, post‑hoc pairwise comparisons of estimated marginal means were conducted using Bonferroni correction to control for multiple testing (*p* = 0.05). For each App, model‑based marginal means and 95% confidence intervals were obtained.

## Results

Figure 1 presents an example of the superimposition of the same IOS scan (IMP_STL) onto the reference dataset (REF_STL) after automatic alignment performed by the four Apps. This example reflects the general visual behavior observed across the dataset: the signed‑distance patterns appear nearly identical among all Apps, whereas slight variations in the color‑coded displays arise from App‑specific color scales and small differences in the computed distance values. Overall, this preliminary visual assessment suggests that the alignment process is broadly comparable across the tested Apps.

**Fig. 2:**
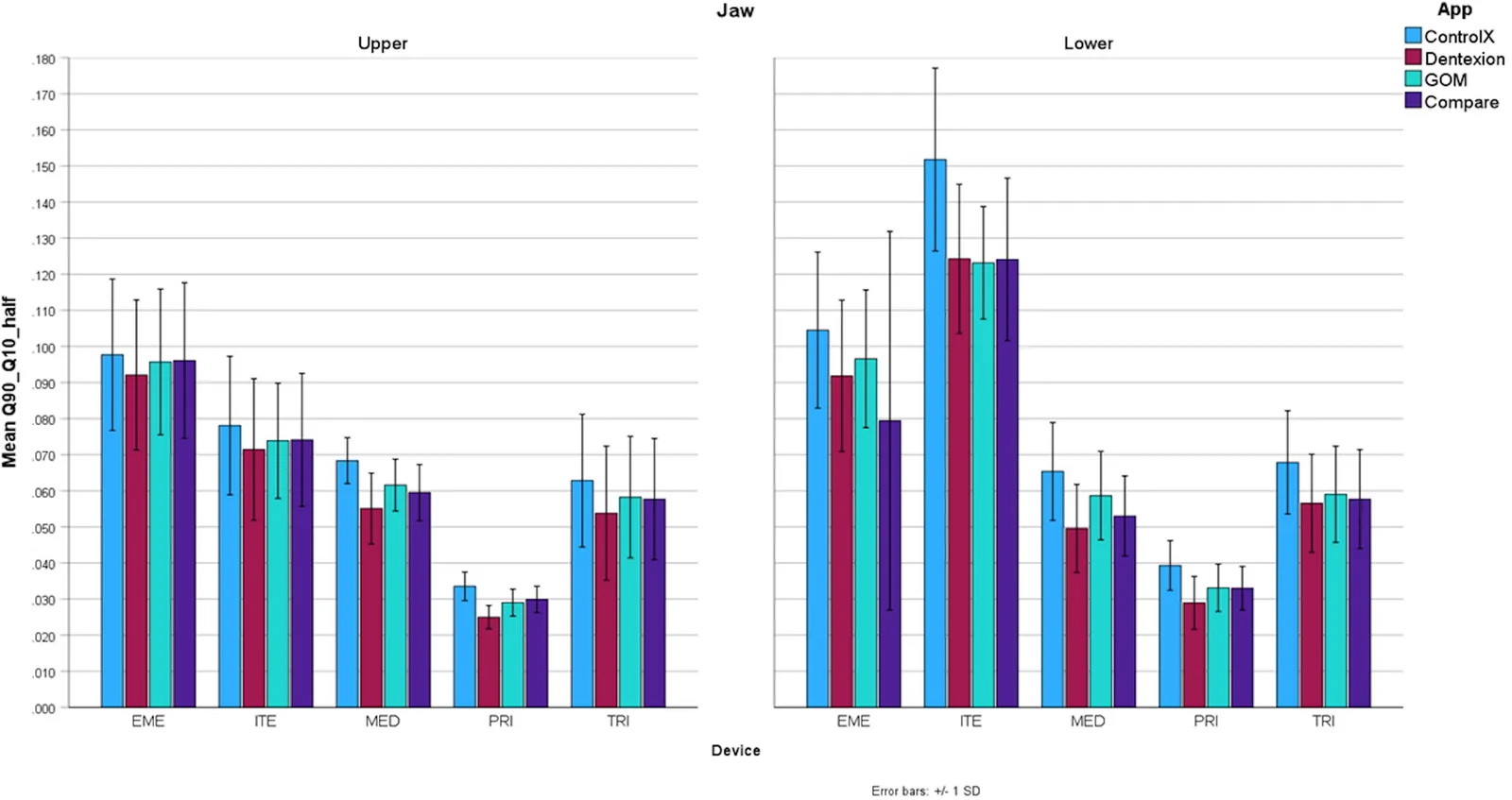
The mean and the standard deviation (SD) for the metric Q90_Q10_half in dependence on Device and App, on the left for the upper jaw reference model and on the right for the lower jaw reference model

**Fig. 3:**
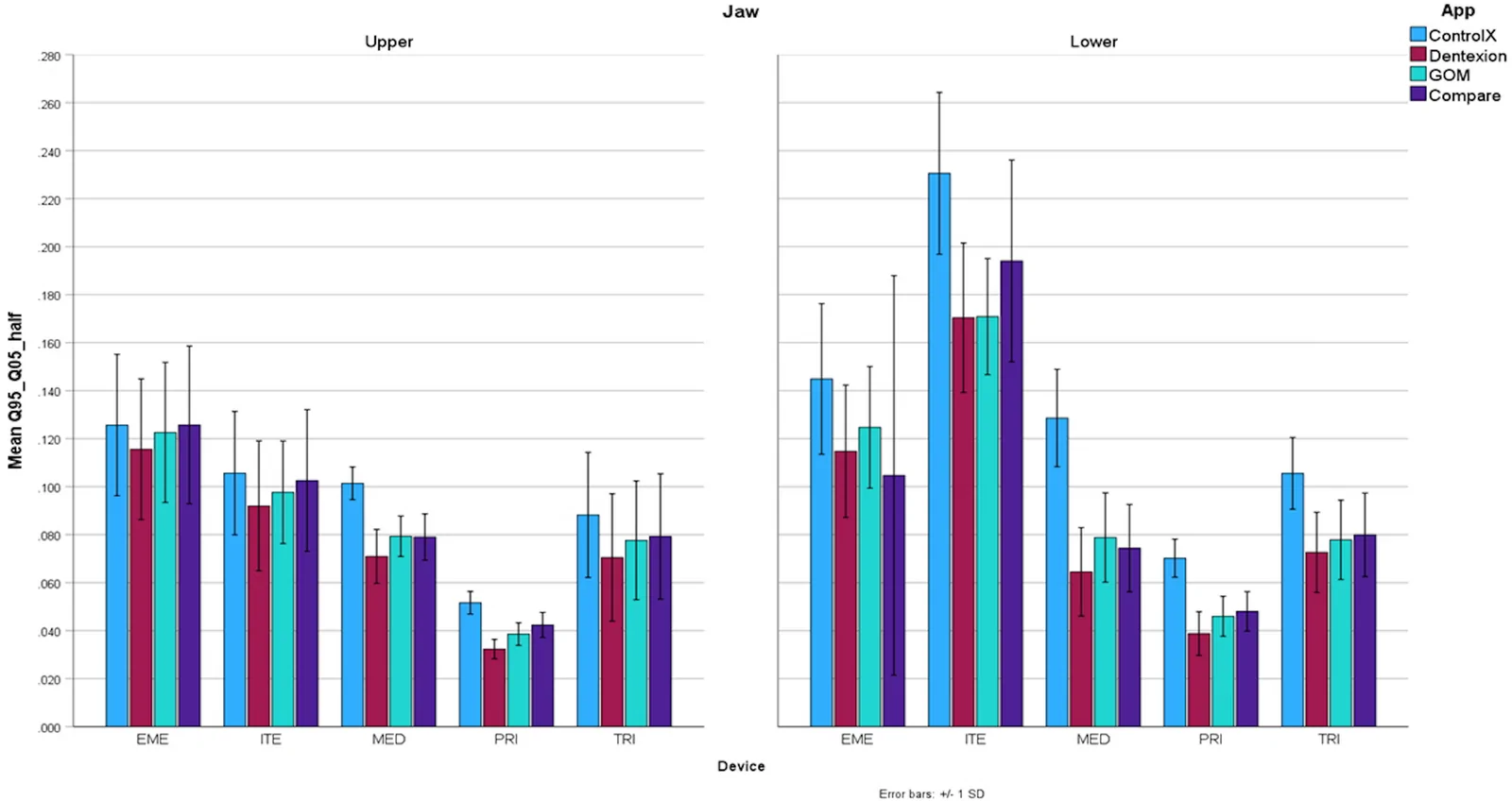
Similar to Fig. 2, the mean and the standard deviation (SD) for the metric Q95_Q05_half in dependence on Device, App and Jaw

The results of the quantitative evaluation are displayed in Figs. 2 and 3 and listed in Tables 1, 2 and 3. In Fig. 2; Table 1, the means and the standard deviations of the quantile metric Q90_Q10_half are shown for each group combination. In general, each App ranks the devices and models in the same order. However, within each group combination, ControlX shows higher values than the other evaluation Apps. The mixed‑effects linear analysis confirms that a significant overall effect of App on Q90_Q10_half after accounting for Device and Jaw (F(3, 391.0) = 4.01, *p* < 0.01). After pairwise comparisons, ControlX yields significant higher adjusted mean values than Dentexion and Compare (Bonferroni‑corrected both *p* < 0.01), while differences among Dentexion, GOM, and Compare were not statistically significant (Table 3). Variance decomposition indicates notable clustering by Device, supporting the use of a mixed‑effects framework for unbiased inference on inter‑App differences.

**Table 1:** Mean and the standard deviation (SD) in mm for the metric Q90_Q10_half from Fig. 1 for the Apps in dependence on Device and Jaw

	Metric	Q90_Q10_half																			
	Device	EME				ITE				MED				PRI				TRI			
	**Jaw**	Upper		Lower		Upper		Lower		Upper		Lower		Upper		Lower		Upper		Lower	
	**[mm]**	Mean	SD	Mean	SD	Mean	SD	Mean	SD	Mean	SD	Mean	SD	Mean	SD	Mean	SD	Mean	SD	Mean	SD
App	**ControlX**	0.098	0.021	0.105	0.022	0.078	0.019	0.152	0.025	0.068	0.006	0.065	0.014	0.034	0.004	0.039	0.007	0.063	0.018	0.068	0.014
	**Dentexion**	0.092	0.021	0.092	0.021	0.071	0.020	0.124	0.021	0.055	0.010	0.050	0.012	0.025	0.003	0.029	0.007	0.054	0.019	0.057	0.014
	**GOM**	0.096	0.020	0.097	0.019	0.074	0.016	0.123	0.016	0.062	0.007	0.059	0.012	0.029	0.004	0.033	0.007	0.058	0.017	0.059	0.013
	**Compare**	0.096	0.022	0.079	0.052	0.074	0.018	0.124	0.023	0.060	0.008	0.053	0.011	0.030	0.004	0.033	0.006	0.058	0.017	0.058	0.014

**Table 2:** Mean and the standard deviation (SD) in mm for the metric Q95_Q05_half from Fig. 2 for the Apps in dependence on Device and Jaw

	Metric	Q95_Q05_half																			
	Device	EME				ITE				MED				PRI				TRI			
	**Jaw**	Upper		Lower		Upper		Lower		Upper		Lower		Upper		Lower		Upper		Lower	
	**[mm]**	Mean	SD	Mean	SD	Mean	SD	Mean	SD	Mean	SD	Mean	SD	Mean	SD	Mean	SD	Mean	SD	Mean	SD
App	**ControlX**	0.126	0.029	0.145	0.031	0.106	0.026	0.231	0.034	0.101	0.007	0.129	0.020	0.052	0.005	0.070	0.008	0.088	0.026	0.106	0.015
	**Dentexion**	0.116	0.029	0.115	0.028	0.092	0.027	0.170	0.031	0.071	0.011	0.065	0.018	0.032	0.004	0.039	0.009	0.071	0.027	0.073	0.017
	**GOM**	0.123	0.029	0.125	0.025	0.098	0.021	0.171	0.024	0.079	0.008	0.079	0.019	0.039	0.005	0.046	0.008	0.078	0.025	0.078	0.017
	**Compare**	0.126	0.033	0.105	0.083	0.103	0.030	0.194	0.042	0.079	0.010	0.074	0.018	0.042	0.005	0.048	0.008	0.079	0.026	0.080	0.017

**Table 3:** Estimated Marginal Mean (EMM) differences and post-hoc Bonferroni‑corrected pairwise differences between Apps from the linear mixed‑effects model for the metrics Q90_10_half and Q95_05_half (fixed effect: App; random effects: Device, Jaw). Significant differences (*p* < 0.05) are indicated with an *

Comparison	EMM Difference Q90_Q10_half [mm]	*p*-value (Adj.)	EMM Difference Q95_Q05_half [mm]	*p*-value (Adj.)
ControlX - Dentexion	0.012	< 0.01*	0.031	< 0.01*
ControlX - GOM	0.008	0.11	0.024	< 0.01*
ControlX - Compare	0.010	< 0.01*	0.022	< 0.01*
Dentexion - GOM	-0.004	1.000	-0.007	0.707
Dentexion - Compare	-0.001	1.000	-0.009	0.327
GOM - Compare	0.002	1.000	-0.002	1.000

The results for the quantile metric Q95_Q05_half are presented in Fig. 3; Table 2. Similarly to the Q90_Q10_half metric, Q95_Q05_half ranks the devices and jaw groups in the same order for each App. However, ControlX shows a distinctly higher value behaviour than the other Apps. Consequently, the linear mixed‑effects model revealed a highly significant effect of App on Q95_05_half (F(3, 390) = 18.07, *p* < 0.001). After adjusting for Device and Jaw variability, ControlX produced significantly higher Q95_05_half values than all other Apps (Bonferroni‑corrected all *p* < 0.01), whereas Dentexion, GOM, and Compare did not differ significantly from each other (Table 3). These results indicate that Q95_05_half is more sensitive to systematic differences between evaluation applications than the Q90_10_half metric.

A post hoc power analysis based on the observed effects of the primary outcome measures indicated statistical powers of approximately 0.85 for Q90_Q10_half and > 0.99 for Q95_Q05_half, indicating that the achieved sample size was sufficient to detect the observed App-related differences.

## Discussion

The hypothesis that evaluation software (Apps) yield comparable quantile‑based deviation metrics has to be rejected. Using a linear mixed‑effects model with App as a fixed effect and random intercepts for Device and Jaw, results show significant differences for both metrics Q90_10_half and Q95_05_half. For Q90_10_half, the magnitude of the inter‑App separation was limited to ControlX versus the two Apps Dentexion and Compare; for Q95_05_half, the separation was larger, with ControlX higher than all comparators.

These findings align with prior studies that software choice and analysis pipeline can systematically alter 3D deviation outcomes, reflecting differences in alignment strategies, outlier handling, sampling density, or point‑to‑point versus point‑to‑surface distance definitions. Studies comparing metrology/evaluation packages (e.g., Geomagic Control X, GOM Inspect, CloudCompare, 3‑matic) have demonstrated statistically significant differences in error metrics in smaller regions (half‑arch, tooth preparation), even when complete‑arch comparisons were less sensitive - indicating that the software layer itself can bias the reported magnitude of deviation metrics [12]. Notably, in that study [12], Geomagic Control X yielded higher or lower RMS values than GOM Inspect depending on the size of the evaluated surface, highlighting a region‑size-by–software interaction; on the other side, CloudCompare and ControlX behave similarly, showing no significant differences. Consistent with our study, Dede et al. (2024) [14] observed a trend - despite not always clinically relevant - for ControlX to produce higher RMS values than Compare. By contrast, another investigation that used different metric (mean deviations at a limited set of points rather than RMS), found ControlX and Compare to be nearly comparable and both lower than GOM, and further argued that the operator influence may be more pronounced when using GOM [13]. Echoing our experience, this suggests that dentistry‑focused superimposition tools - particularly those enabling automated, anatomy‑aware segmentation (e.g., automated tooth segmentation) and more constrained ROIs like Dentexion and Compare - can help reduce operator‑introduced variability. Finally, when contrasted with open‑source platforms, Mourouzis (2025) [15] reported significantly lower RMS values with Geomagic Control X than with MeshLab or CloudCompare (the latter two performing similarly). These differences likely reflect distinct algorithmic implementations and design priorities of the respective programs, many of which originated for non‑dental applications and may not be optimized for dental surface characteristics or clinical reporting conventions.

These App effects are also algorithmically plausible: the initial and best‑fit alignment (e.g., ICP variants), correspondence rules, and outlier rejection steps are known to impact measured deviations. Methodological work has shown that alignment choices (reference vs. best‑fit vs. landmark‑based) materially affect translation/rotation error and propagate into commonly reported metrics (profilometric/volume/surface change), with “reference‑based” approaches typically reducing bias relative to best‑fit in dental models [21]. More broadly, the ICP family of algorithms and their variants produce different convergence behaviors and sensitivity to local minima, outliers, and sampling - further explaining why different Apps, with different implementations and defaults, may yield different absolute values [22].

An important empirical observation from the result data is that, although absolute quantile values shift by App, the relative ordering of Device and Jaw remains stable (e.g., if PRI < MED for ControlX, the same PRI < MED relationship holds for Dentexion, GOM, and Compare). Two features of the analysis help explain this: First, the mixed‑model ICCs (device ICC ≈ 0.56 for Q90_10_half and ≈ 0.55 for Q95_05_half) indicate that Device accounts for a large fraction of structured variability. Thus, device‑specific baseline levels dominate the scale on which App differences operate. Second, a linear App effect is estimated as a percentage shift common to all devices and jaws. In other words, Apps behave like calibration offsets layered on top of device‑ and jaw‑specific baselines; such offsets preserve the ranking among devices and jaws, even if the absolute values differ by App. This interpretation is consistent with metrology literature showing that different alignment/inspection pipelines can bias the magnitude of deviations without necessarily altering between‑platform rank order, particularly when the dominant source of variance lies at the platform level [23].

At the same time, the prominent device effect observed in this study is consistent with numerous scanner‑level accuracy studies - both in vitro and in vivo - that report between‑scanner differences in trueness/precision across arches and implant scenarios, regardless of the analysis environment. While methods vary (e.g., Control X pipelines, open‑source workflows using CloudCompare/Meshmixer), the common conclusion is that scanner hardware and acquisition workflow are major determinants of the deviation landscape; analysis software then modulates the measured magnitude [24, 25]. 

This study was based on in vitro scan data. In principle, the software algorithms used for superimposition and deviation analysis are independent of whether in vitro or in vivo datasets are analyzed. However, in vivo scans may contain a higher frequency of outliers, artifacts, and scan voids, which could affect the superimposition and distance-calculation processes. Provided that such defects represent only a small proportion of the overall scan surface, their influence on the resulting metrics is expected to be limited. Nevertheless, this potential effect should be considered when interpreting the findings and warrants further in vivo studies in the future.

Across the cited literature [12–15, 24, 25], a clear shift is emerging toward the use of open‑source and dentistry‑focused evaluation software. These platforms offer two major advantages: they reduce operator dependency by incorporating automated analytical steps, and they lower financial barriers compared with industrial metrology software. The results from our study, particularly with Dentexion and Compare, reinforce this trend by demonstrating that such tools can deliver consistent, reliable outputs while also improving user‑friendliness. One of the central strengths of these dental‑specific solutions is their ability to perform automated tooth segmentation, which ensures robust correspondence‑point identification during both the best‑fit/ICP alignment process and the subsequent point-to-surface and surface‑to‑surface deviation calculations. This capability may offer the possibility to enable rapid accuracy checks of IOS devices in clinical settings and to support a variety of ongoing digital monitoring tasks.

Looking ahead, future research should explore more fully the analytical opportunities unlocked by segmented‑tooth information during alignment. For example, software could automatically highlight regions exhibiting disproportionately high deviations, facilitating the detection of systematic deformation patterns. Beyond surface deviation metrics alone, segmentation also makes it possible to extract tooth‑level geometric parameters, such as translational or rotational differences, that may offer more intuitive measures of accuracy. A simple example would be the automated computation of the inter‑molar distance and its variation across repeated scans.

Finally, extending the monitoring and diagnostic analyses to clinical datasets under in vivo scanning conditions will be essential to determine whether any App‑associated offsets remain stable when confronted with the complexities of clinical environments - such as saliva, soft tissue movement, reflective surfaces, and other intraoral variables. Such efforts will improve the external validity and generalizability of future superimposition workflows.

## Summary

This study evaluated how four different analysis software programs (Apps) influence digital accuracy metrics derived from intraoral scanner (IOS) data. The key findings are as follows:

- For the Q90_Q10_half metric, Geomagic Control X produced significantly higher values than both Dentexion and Compare, while no statistically significant differences were observed among Dentexion, GOM Inspect, and Compare.
- For the Q95_05_half metric, Control X again yielded significantly higher values than all other Apps, whereas Dentexion, GOM Inspect, and Compare remained statistically indistinguishable from one another.
- The ranking of IOS devices was not affected by the choice of evaluation software, indicating that while absolute deviation values may shift depending on the App, the relative performance hierarchy of the scanners remains stable.
- Dental‑focused evaluation Apps, which incorporate higher levels of automation and task‑specific features (e.g., automated tooth segmentation), may represent a promising and user‑friendly alternative to industrial metrology software in future.

## Conclusion

Across two quantile‑range metrics, Apps yield systematically different values, with ControlX consistently higher, especially for Q95_05_half, while device rankings remain stable across Apps. These results confirm that evaluation software is an additional factor in digital accuracy studies and should be more standardized in analyses and multi‑center comparisons.

## Data Availability

The data supporting the findings of this study are available from the corresponding author upon reasonable request.
